# Supplementary material for: Quantitative detection and prognostic value of antibodies against M-type phospholipase A2 receptor and its cysteine-rich ricin domain and C-type lectin domains 1 and 6-7-8 in patients with idiopathic membranous nephropathy
Source: PLoS One. 2024 Feb 22;19(2):e0298269. doi: 10.1371/journal.pone.0298269 (PMC10883571; doi:10.1371/journal.pone.0298269)
Supplement: S1 Checklist — (DOCX) [file pone.0298269.s001.docx]

STROBE Statement—checklist of items that should be included in reports of observational studies

|  | Item No. | Recommendation | Page  No. | Relevant text from manuscript |
| --- | --- | --- | --- | --- |
| **Title and abstract** | 1 | (*a*) Indicate the study’s design with a commonly used term in the title or the abstract | 1 | time-resolved fluorescence immunoassay |
|  |  | (*b*) Provide in the abstract an informative and balanced summary of what was done and what was found | 2-3 | Abstract |
| Introduction | | | |  |
| Background/rationale | 2 | Explain the scientific background and rationale for the investigation being reported | 3-4 | Seitz-Polski et al. suggested that an analysis of PLA2R epitopes by characterization and spreading is a powerful tool for monitoring disease severity and stratifying patients by renal prognosis (Fig. 1). Subsequently, Reinhard et al. [6]found that CTLD8 is also an epitope of PLA2R. Therefore, to evaluate the clinical value of epitope spreading in this study, we used highly sensitive time-resolved fluorescence immunoassay (TRFIA) and aimed to quantitatively detect changes in the concentrations of different antibodies against epitopes of PLA2R in patients with IMN before and after treatment. |
| Objectives | 3 | State specific objectives, including any prespecified hypotheses | 3 | the prognosis of a disease can be predicted using epitope spreading |
| Methods | | | |  |
| Study design | 4 | Present key elements of study design early in the paper |  |  |
| Setting | 5 | Describe the setting, locations, and relevant dates, including periods of recruitment, exposure, follow-up, and data collection | 4-5 | 2.2 Sera and patients |
| Participants | 6 | (*a*) *Cohort study*—Give the eligibility criteria, and the sources and methods of selection of participants. Describe methods of follow-up  *Case-control study*—Give the eligibility criteria, and the sources and methods of case ascertainment and control selection. Give the rationale for the choice of cases and controls  *Cross-sectional study*—Give the eligibility criteria, and the sources and methods of selection of participants | 4-5 | 2.2 Sera and patients |
|  |  | (*b*) *Cohort study*—For matched studies, give matching criteria and number of exposed and unexposed  *Case-control study*—For matched studies, give matching criteria and the number of controls per case | 4-5 | 2.2 Sera and patients |
| Variables | 7 | Clearly define all outcomes, exposures, predictors, potential confounders, and effect modifiers. Give diagnostic criteria, if applicable | 6 | 2.3.4 Definitions and calculations |
| Data sources/ measurement | 8* | For each variable of interest, give sources of data and details of methods of assessment (measurement). Describe comparability of assessment methods if there is more than one group | 4-5 | 2.1 Chemicals and instrumentation  2.3 PLA2R-, CysR-, CTLD1-, and CTLD6-7-8-specific TRFIAs |
| Bias | 9 | Describe any efforts to address potential sources of bias | 6 | 2.4 Statistical analyses |
| Study size | 10 | Explain how the study size was arrived at | 4 | 2.2 Sera and patients |

Continued on next page

| Quantitative variables | 11 | Explain how quantitative variables were handled in the analyses. If applicable, describe which groupings were chosen and why | 6 | 2.4 Statistical analyses |
| --- | --- | --- | --- | --- |
| Statistical methods | 12 | (a) Describe all statistical methods, including those used to control for confounding | 6 | 2.4 Statistical analyses |
|  |  | (b) Describe any methods used to examine subgroups and interactions |  |  |
|  |  | (c) Explain how missing data were addressed |  | Missing data were represented as censored data |
|  |  | (d) Cohort study—If applicable, explain how loss to follow-up was addressed  Case-control study—If applicable, explain how matching of cases and controls was addressed  Cross-sectional study—If applicable, describe analytical methods taking account of sampling strategy |  |  |
|  |  | (*e*) Describe any sensitivity analyses | 6 | 2.4 Statistical analyses |
| Results | | | | |
| Participants | 13* | (a) Report numbers of individuals at each stage of study—eg numbers potentially eligible, examined for eligibility, confirmed eligible, included in the study, completing follow-up, and analysed | 6 | Twenty-five patients with IMN were evaluated and followed up for six months, during which time they were treated with immunosuppressive therapy. The patients were divided into a remission group (including PR and CR, n = 13) and a non-remission group (n = 12) depending on their clinical conditions at the last follow-up. |
|  |  | (b) Give reasons for non-participation at each stage |  |  |
|  |  | (c) Consider use of a flow diagram |  |  |
| Descriptive data | 14* | (a) Give characteristics of study participants (eg demographic, clinical, social) and information on exposures and potential confounders | 7-8 | Table 1 |
|  |  | (b) Indicate number of participants with missing data for each variable of interest | No |  |
|  |  | (c) *Cohort study*—Summarise follow-up time (eg, average and total amount) | 6 | Twenty-five patients with IMN were evaluated and followed up for six months, during which time they were treated with immunosuppressive therapy. |
| Outcome data | 15* | *Cohort study*—Report numbers of outcome events or summary measures over time | 6 | The patients were divided into a remission group (including PR and CR, n = 13) and a non-remission group (n = 12) depending on their clinical conditions at the last follow-up. |
|  |  | *Case-control study—*Report numbers in each exposure category, or summary measures of exposure |  |  |
|  |  | *Cross-sectional study—*Report numbers of outcome events or summary measures |  |  |
| Main results | 16 | (*a*) Give unadjusted estimates and, if applicable, confounder-adjusted estimates and their precision (eg, 95% confidence interval). Make clear which confounders were adjusted for and why they were included |  |  |
|  |  | (*b*) Report category boundaries when continuous variables were categorized | 7 | Table 1 |
|  |  | (*c*) If relevant, consider translating estimates of relative risk into absolute risk for a meaningful time period |  |  |

Continued on next page

| Other analyses | 17 | Report other analyses done—eg analyses of subgroups and interactions, and sensitivity analyses | No |  |
| --- | --- | --- | --- | --- |
| Discussion | | | | |
| Key results | 18 | Summarise key results with reference to study objectives | 11 | Therefore, the simultaneous analyses of specific IgG and IgG4 antibodies against PLA2R and its CysR, CTLD1, and CTLD6-7-8 epitopes through highly sensitive quantitative assays can explain the different views in previous reports. Epitope spreading does not play a significant role in evaluating the therapeutic effect if only the numbers of identifiable epitopes are qualitatively determined by specific IgG antibodies. However, the highly sensitive quantitative detection of changes in the concentrations of specific IgG4 antibodies against PLA2R and its CysR, CTLD1, and CTLD6-7-8 epitopes can be a good prognostic tool. Therefore, a quantitative detection rather than a qualitative analysis of epitope spreading is preferred. |
| Limitations | 19 | Discuss limitations of the study, taking into account sources of potential bias or imprecision. Discuss both direction and magnitude of any potential bias | 11 | This study has certain limitations. Given that the follow-up results were evaluated after 6 months of immunosuppressive therapy, data at this point indicated that the concentrations of IgG4 antibodies against PLA2R and its epitopes had largely returned to normal in patients from the remission group. However, it is possible that the concentrations of IgG4 antibodies against PLA2R and its epitopes had returned to normal earlier than 6 months, which may be suitable for accurate evaluation. |
| Interpretation | 20 | Give a cautious overall interpretation of results considering objectives, limitations, multiplicity of analyses, results from similar studies, and other relevant evidence | 11 | In conclusion, the combined quantitative detection of specific IgG and IgG4 antibodies against PLA2R and its CysR, CTLD1, and CTLD6-7-8 epitopes can identify epitope spreading in patients with IMN, and the detection of specific IgG4 antibodies can be more used for prognosis. |
| Generalisability | 21 | Discuss the generalisability (external validity) of the study results | No |  |
| Other information | |  | | |
| Funding | 22 | Give the source of funding and the role of the funders for the present study and, if applicable, for the original study on which the present article is based | 14 | This study was supported by the Social Development Fund of Zhejiang Province [No. LGF20H200008]; Chinese National Natural Science Foundation [No. 82172336,81672083,82070730]; Key Research and Development Project of Zhejiang Province (No. 2022C03118), the Natural Science Foundation of Zhejiang Province(LQ23H050005), Key Research and Development Project of Hangzhou (No. 202004A23),Precision medicine key project of Wuxi Health Commission [No. J202001], theTop Talent Support Program for young and middle-aged people of Wuxi Health Committee [HB2020008],Wuxi Medical Innovation Team Project [CXTD2021010],Specialized Disease Cohort of Wuxi Medical Center of Nanjing Medical University(WMCC202316). |

*Give information separately for cases and controls in case-control studies and, if applicable, for exposed and unexposed groups in cohort and cross-sectional studies.

**Note:** An Explanation and Elaboration article discusses each checklist item and gives methodological background and published examples of transparent reporting. The STROBE checklist is best used in conjunction with this article (freely available on the Web sites of PLoS Medicine at http://www.plosmedicine.org/, Annals of Internal Medicine at http://www.annals.org/, and Epidemiology at http://www.epidem.com/). Information on the STROBE Initiative is available at www.strobe-statement.org.
